# Histone Acetylation Promotes Neutrophil Extracellular Trap Formation

**DOI:** 10.3390/biom9010032

**Published:** 2019-01-18

**Authors:** Hussein J. Hamam, Meraj A. Khan, Nades Palaniyar

**Affiliations:** 1 Program in Translational Medicine, Peter Gilgan Centre for Research and Learning, The Hospital for Sick Children, Toronto, ON M5G 0A4, Canada; hussein.hamam@sickkids.ca (H.J.H.); meraj.khan@sickkids.ca (M.A.K.); 2 Department of Laboratory Medicine and Pathobiology, Faculty of Medicine, University of Toronto, Toronto, ON M5S 1A8, Canada; 3 Institute of Medical Sciences, Faculty of Medicine, University of Toronto, Toronto, ON M5S 1A8, Canada

**Keywords:** neutrophils, NETosis, histone acetylation, histone decondensation, histone deacetylase inhibitors

## Abstract

Neutrophils undergo a unique form of cell death to generate neutrophil extracellular traps (NETs). It is well established that citrullination of histones (e.g., CitH3) facilitates chromatin decondensation during NET formation (NETosis), particularly during calcium-induced NETosis that is independent of nicotinamide adenine dinucleotide phosphate (NADPH) oxidase (NOX) activation. However, the importance of other forms of histone modifications in NETosis has not been established. We considered that acetylation of histones would also facilitate NETosis. To test this hypothesis, we induced NOX-dependent NETosis in human neutrophils with phorbol myristate acetate or lipopolysaccharide (from *Escherichia coli* 0128), and NOX-independent NETosis with calcium ionophores A23187 or ionomycin (from *Streptomyces conglobatus*) in the presence or absence of two pan histone deacetylase inhibitors (HDACis), belinostat and panobinostat (within their half maximal inhibitory concentration (IC50) range). The presence of these inhibitors increased histone acetylation (e.g., AcH4) in neutrophils. Histone acetylation was sufficient to cause a significant increase (~20%) in NETosis in resting neutrophils above baseline values. When acetylation was promoted during NOX-dependent or -independent NETosis, the degree of NETosis additively increased (~15–30%). Reactive oxygen species (ROS) production is essential for baseline NETosis (mediated either by NOX or mitochondria); however, HDACis did not promote ROS production. The chromatin decondensation step requires promoter melting and transcriptional firing in both types of NETosis; consistent with this point, suppression of transcription prevented the NETosis induced by the acetylation of histones. Collectively, this study establishes that histone acetylation (e.g., AcH4) promotes NETosis at baseline, and when induced by both NOX-dependent or -independent pathway agonists, in human neutrophils. Therefore, we propose that acetylation of histone is a key component of NETosis.

## 1. Introduction

Neutrophils are terminally differentiated innate immune cells (60–70% of all white blood cells) that possess different strategies to fight pathogens, including degranulation, oxidative burst and phagocytosis, and neutrophil extracellular trap (NET) formation [1,2,3]. In response to certain inflammatory stimuli (e.g., phorbol 12-myristate 13-acetate (PMA), lipopolysaccharide (LPS), A23187, ionomycin), these cells can undergo a unique form of programmed cell death to generate NETs [4,5]. Neutrophil extracellular traps are web-like structures that are made of decondensed chromatin coated with cytotoxic peptides. During NET formation (NETosis), increased intracellular levels of reactive oxygen species (ROS) promote pathway-specific kinase activation, promoter melting, transcriptional firing, extensive chromatin decondensation and eventual NET release [3,6,7]. To date, studies have shown that NETs are formed through two major pathways: NOX-dependent and -independent pathways [1,3]. The former requires the NOX-derived ROS whereas the latter requires mitochondrial ROS (mROS) production [1,3]. At later stages of NETosis, granular enzymes such as myeloperoxidase (MPO) and elastases coat the decondensed histones before being released as NETs.

Histone modifications could regulate chromatin decondensation and subsequent NET formation. The citrullination of histones is considered as an important modification that facilitates chromatin decondensation during NETosis. Increased intracellular calcium levels activate peptidylarginine deiminase 4 (PAD4), which rapidly translocate into the nucleus and convert the arginine present on histones (e.g., H3) into citrulline [1,8]. The loss of the positive charge of the modified histone allows local decompaction of chromatin. Citrullination of histone H3 (CitH3) has been shown to occur preferentially at promoter regions, and hence, could facilitate transcriptional firing and rapid chromatin decondensation [3,8]. Recent studies, published from our lab and others show that CitH3 formation is a hallmark of NOX-independent NETosis, but not NOX-dependent NETosis [1,9,10]. However, CitH3 is not the only post-translational modification; acetylation could also decondense histones and increase gene expression [11,12]. Yet, the relevance of histone acetylation in NETosis is unknown.

Histone acetylation (e.g., acetylated histone H4, AcH4), neutralizes the positive charge. Hence, this alters the histone interaction with the negatively charged DNA, which allows transcription factors to bind to the promoter regions and initiate transcription [13,14]. Conversely, the removal of acetyl groups by histone deacetylase complex (HDAC) results in the compaction of the chromatin and inhibition of transcription [14]. Neutrophils express 18 different HDACs, hence, these enzymes could promote effective chromatin compaction [15,16]. Currently, there are 5 classes of HDAC inhibitors (HDACis) available [17,18]. These are either class specific or pan-deacetylase (pan-DAC) inhibitors, which inhibit most of HDACs; 3 of them are approved for clinical use, including belinostat and panobinostat. The HDACis prevent histone deacetylation, and hence, could promote histone acetylation, transcription and chromatin decondensation for NET formation.

We hypothesized that histone acetylation promotes NETosis. To test this, we used two pan HDAC inhibitors belinostat and panobinostat, and tested histone acetylation, ROS production, transcription and NETosis at baseline and with the induction of NOX-dependent and independent pathways. By using primary neutrophils of healthy donors, our studies show that HDACis promote histone acetylation and spontaneous NETosis (~20%) above baseline. AcH4 formation also increases during NETosis induced by HDACis, which also have an additive effect of ~15–30% on both NOX-dependent and -independent NETosis. In addition, HDACis increase NETosis without significantly altering ROS generation either by NOX or mitochondria. Nevertheless, the inhibition of transcription suppressed acetylation-induced NETosis. This is consistent with the fact that transcriptional firing, which is downstream of histone acetylation, is required for NETs formation [3]. Therefore, for the first time, we show that histone acetylation promotes NET formation by facilitating chromatin decondensation. This finding would be useful for delineating molecular mechanisms of NETosis, and may be helpful for understanding the alterations in NETosis during autoimmune diseases and in clinical conditions in which these HDAC inhibitors are used for suppressing cell proliferation (e.g., cancer treatments) [19,20].

## 2. Materials and Methods

### 2.1. Research Ethics Board Approval

The study protocol for using human blood samples was approved by the ethics committee of The Hospital for Sick Children, Toronto (No. 1000020217). All methods, including healthy human volunteer recruitment for blood donation, were performed in accordance with the ethics committee guidelines. Signed informed consents were provided by all the volunteers participating in this study prior to the blood donation.

### 2.2. Human Peripheral Blood Neutrophil Isolation

Neutrophils from healthy male donors were used in the study. Donors with eosinophils or either a too low or too high neutrophil count were excluded from the study. This protocol usually yields 1–1.5 million neutrophils per mL of blood. A volume of 40–60 mL of peripheral blood was drawn from healthy donors into K2 EDTA blood collection tubes (Becton, Dickinson and Co., Franklin Lakes, NJ, USA) at the nursing station of the hospital. PolymorphPrep^TM^ (Axis-Shield, Oslo, Norway) was used for purifying neutrophils from peripheral blood by following the manufacturer’s instructions with minor modifications. An equal volume of blood was layered over PolymorphPrep solution and was then centrifuged at 600 × *g* for 35 min without any brakes. After centrifugation, the polymorphonuclear neutrophil layer was collected and a washing solution (0.425% (*w*/*v*) NaCl with 10 mM HEPES) was used for eliminating residues of PolymorphPrep. To purify neutrophils from the residual red blood cells (RBCs), cells were washed twice with hypotonic solution (0.2% (*w*/*v*) NaCl) for 30 s, followed by adding an equal volume of 1.6% (*w*/*v*) NaCl solution with 20 mM HEPES buffer to obtain the isotonic condition. The cells were then washed twice to eliminate RBCs debris and soluble components. RPMI 1640 medium (Invitrogen, Carlsbad, CA, USA) supplemented with 10 mM HEPES buffer was used for re-suspending neutrophils. Cell density was quantified using a hemocytometer, and Cytospin preparations were used to check the purity of the neutrophils. Neutrophil preparations with >95–98% were used in all the experiments.

### 2.3. Sytox Green NETosis Assay

Sytox Green, a cell-impermeable DNA binding dye (Life Technologies, Carlsbad, CA, USA), was used for estimating NETosis kinetics. A volume of 100 μL media (RPMI 1640 medium supplemented with 10 mM HEPES) containing 50,000 neutrophils and 5 μM Sytox Green were seeded into 96-well black clear-bottom plates. A volume of 5 µL of HDACis (final concentrations: 62.5, 125, and 250 nM belinostat (PXD-101); 10, 20, and 40 nM panobinostat (LBH589)) or actinomycin D (Act-D; final concentrations: 2.5 and 5 µM) was added to respective wells with controls (RMPI + neutrophils only) for 30 min at 37 °C and 5% (*v*/*v*) CO_2_. 5 µL agonists (final concentrations: 25 nM PMA; 4 μM A23187; 5 μg/mL LPS (from *E. coli* 0128); 5 μM Ionomycin, (unless otherwise stated)) were then added and placed at 37 °C and 5% (*v*/*v*) CO_2_ incubator. Sytox Green fluorescence intensities were measured every 60 min for up to 4 h using a fluorescence plate reader (504 nm excitation, 523 nm emission, POLARstar OMEGA, BMG Labtech, Guelph, ON, Canada). The plates were briefly taken out for ~2–5 min, of the incubator for the readings. To calculate the NETotic index (% of Sytox Green accessible total DNA), the baseline green fluorescence at time 0-min was subtracted from the fluorescence at each time point and was then divided by the fluorescence values of 100% NET formation obtained by lysing the cells with 0.5% (*v*/*v*) Triton X-100 (240 min time point).

### 2.4. DHR123, DCFDA and MitoSOX Plate Reader Assays (NOX- and Mitochondrial-Mediated ROS Analyses)

A volume of media containing 100,000 neutrophils and 20 μM dihydrorhodamine 123 (DHR123, Thermo Fisher Scientific, Waltham, MA, USA) were incubated at 37 °C and 5% (*v*/*v*) CO_2_ incubator for 15 min. The cells were then centrifuged for 10 min (400 × *g*; with 9 acceleration and 9 deceleration ramp) and were washed with an equal amount of media. Neutrophil-containing solutions (100 μL RPMI) were seeded into 96-well black clear-bottom plates and 1 µM diphenyleneiodonium (DPI) or 200 µM MitoTEMPO (Sigma-Aldrich, Oakville, ON, Canada) and HDACis (belinostat and panobinostat) were added to respective wells with controls (RMPI + neutrophils only) for 60 min and 30 min, respectively, at 37 °C and 5% (*v*/*v*) CO_2_. Then, 5 µL agonists (final concentrations: 25 nM PMA; 4 μM A23187; 5 μg/mL LPS from *E. coli* 0128; 5 μM Ionomycin) were added and placed at 37 °C and 5% (*v*/*v*) CO_2_ incubator. Fluorescence was measured every 10 min in the first 30 min and then every 30 min up until 90 min by using a fluorescence plate reader (507 nm excitation, 529 nm emission, POLARstar OMEGA, BMG Labtech). Relative fluorescence units (RFU) were calculated by subtracting the fluorescence at each time point from the baseline fluorescence at time 0-min.

The 2’,7’-dichlorofluorescin diacetate (DCFDA) and MitoSOX Red (Thermo Fisher Scientific) plate reader assays were performed similarly to DHR assay with the following exceptions: 15 μM DCFDA and 5 μM MitoSOX red were incubated at 37 °C and 5% (*v*/*v*) CO_2_ incubator for 15 min (no washing) and measured by fluorescence plate reader (DCFDA assay: 429 nm excitation, 520 nm emission; MitoSOX assay: 510 nm excitation, 580 nm emission).

### 2.5. Fluorescence Confocal Imaging

A volume of 100 µL of media containing 100,000 neutrophils were seeded into 12-well chamber slides. 5 µL of HDACi (250 nM belinostat; 20 nM panobinostat) and/or NETotic agonists (25 nM PMA; 4 μM A23187; 5 μg/mL LPS from *E. coli* 0128; 5 μM Ionomycin) were then added to respective wells with controls (RMPI + neutrophils only) and incubated for 120 min at 37 °C and 5% (*v*/*v*) CO_2_. Paraformaldehyde (4%, *w*/*v*) was used for fixing neutrophils and NETs for 30 min, washed and then permeabilized with 0.1% Triton-X 100 for 15 min at room temperature. Bovine serum albumin (BSA) (5%, *w*/*v*) was used for blocking unspecific binding for 60 min at room temperature. After washing with PBS, the specimen was incubated with primary antibodies: mouse anti-MPO antibody (ab25989, Abcam; 1:500 dilution) was used for staining MPO (with secondary antibody conjugated with a green fluorescence Alexa Fluor 488 dye; 1:1000 dilution; Thermo Fisher Scientific), while rabbit anti-CitH3 antibody (ab5103, Abcam; 1:500 dilution) or rabbit anti-histone H4 (acetyl K5) antibody (ab51997, Abcam, 1:1000) was used for detecting the presence of CitH3 or acetylated H4K5 (H4K5ac), respectively (with secondary antibody conjugated with a far-red fluorescence dye Alexa Fluor 647; 1:1000 dilution; Thermo Fisher Scientific). DNA was stained with DAPI (1:100 dilution). After treating with the secondary antibody, slides were washed and mounted by glass coverslips (Fisher Scientific) with anti-fade fluorescent mounting medium (Dako, Carpinteria, CA, USA). The images were then taken using an Olympus IX81 inverted fluorescence microscope with a Hamamatsu C9100-13 back-thinned EM-CCD camera and Yokogawa CSU × 1 spinning disk confocal scan head (Olympus Canada Inc., Richmond Hill, ON, Canada) with Spectral Aurora Borealis upgrade, four separate diode-pumped solid-state laser lines (405, 491, 561, and 642 nm; Spectral Applied Research, Richmond Hill, ON, Canada). The images were taken at 40 × /0.95 magnification and processed by Volocity software (version 6.3, Cell Imaging Perkin-Elmer; Quorum Technologies Inc., Puslinch, ON, Canada).

### 2.6. Western Blot

Tubes containing 1 × 10^6^ cells were treated with 5 µL of HDACis (250 nM belinostat; 20 nM panobinostat), NETotic agonists (25 nM PMA; 4 μM A23187; 5 μg/mL LPS from *E. coli* 0128; 5 μM Ionomycin) and/or ultraviolet (UV) irradiation (0.24 J/cm^2^) for 90 min at 37 °C and 5% (*v*/*v*) CO_2_. Cells were then cooled on ice for 10 min. Neutrophils were lysed using the lysis buffer containing RIPA lysis buffer (Millipore, Etobicoke, ON, Canada) containing 1 mM PMSF, 1 mM sodium orthovanadate, 1 mM sodium fluoride, 1 mg/mL aprotinin, 1 mg/mL leupeptin, 1 mg/mL pepstatin, and a protease inhibitor cocktail tablet per 5 mL (Roche Diagnostics, Laval, QC, Canada), DNase I (Invitrogen) and a phosphatase inhibitor cocktail tablet per 10 mL (Roche). Samples were vortexed for 10 s to mix the solution and incubated for 30 min at 37 °C and 5% (*v*/*v*) CO_2_ and then sonicated 3 times for 30-s intervals. The bicinchoninic acid (BCA) protein assay kit (Thermo Fisher Scientific) was used to ensure the same amount of total protein of each sample was used. Each sample was mixed with reducing sample loading buffer (20% (*v*/*v*) glycerol, 2% (*v*/*v*) beta-mercaptoethanol, 4% (*w*/*v*) SDS, 0.130 M Tris, Bromophenol Blue (1 mg/100 mL), pH 6.8). Samples were heated at 99 °C for 5 min prior to loading into SDS-PAGE gel. Samples were run on a 4–20% Mini-PROTEAN^®^ TGX™ precast protein gels (Bio-RAD Laboratories, Mississauga, ON, Canada) at 120 V for 60 min to separate protein by size. Proteins were then transferred to nitrocellulose membranes by wet transfer. The empty spaces on the membranes were then blocked with 5% (*w*/*v*) BSA in 0.1% (*v*/*v*) TBST for 1 h at room temperature. All primary antibodies were dissolved in 1% (*w*/*v*) BSA in 0.1% (*v*/*v*) TBST and incubated with membranes overnight at 4 °C followed by 3 washes with 0.1% PBST for 30 min. Primary antibodies used were: anti-GADPH (2118, Cell Signaling Technology, Whitby, ON, Canada) rabbit mAb at 1:1000; anti-histone H4K5ac (ab51997, Abcam) rabbit mAb at 1:1000; anti-cleaved caspase-3 (Asp175, Cell Signaling) rabbit mAb at 1:1000. The membranes were then incubated in the secondary antibody solution (1% (*w*/*v*) BSA in 0.1% (*v*/*v*) TBST) for 60 min and then washed 3 times with 0.1% (*v*/*v*) PBST for 30 min. The secondary antibody used was: anti-rabbit IgG-HRP (Cell Signaling) at 1:5000. The protein intensity was determined by enhanced chemiluminescent reagents, and the blots were imaged in Li-Cor Odyssey FC Imaging System and densitometry analysis of the images performed using Image Studio software (LI-COR Biotechnology, Lincoln, NE, USA). The H4K5ac protein bands were normalized to the GAPDH bands.

### 2.7. Statistical Analyses

All data are presented as mean ± standard error of the mean (SEM) in line graphs. In box plots, each data point is presented. The mean is indicated with “+”, and the full data spread is indicated with lines and boxes are marked with median and upper and lower interquartile ranges. GraphPad Prism statistical analysis software (Version 8.00 for Windows, San Diego, CA, USA) was used for performing the statistical analysis by One-way ANOVA with a Dunnett or Tukey test, where appropriate. A Dunnett post-test was used for comparing a fixed value of 1 with respective treatment conditions. A mean difference with a *p*-value of ≤ 0.05 was considered to be statistically significant.

## 3. Results

### 3.1. HDAC Inhibitors Promote Histone Acetylation

Histone acetylation results in chromatin relaxation and subsequent increase in gene transcription [13,14]. Therefore, we questioned whether HDACis have the potential to promote histone acetylation in neutrophils for promoting NETosis. To test this point, we performed Western blotting and immunofluorescence assays using neutrophils treated with belinostat, panobinostat, and/or NETotic agonists and then stained against DAPI and H4K5ac (AcH4).

**Figure 1:**
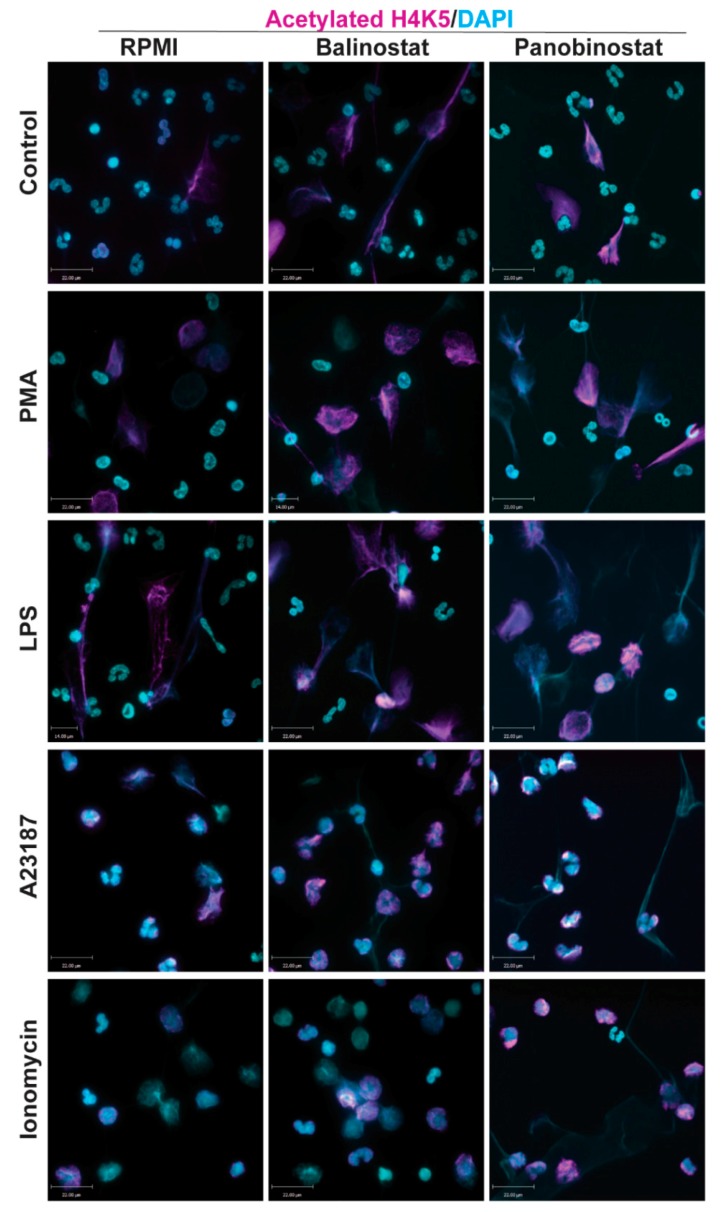
Confocal microscopy images showing histone deacetylase inhibitors (HDACi) promote histone acetylation. Neutrophils were treated with negative control (RPMI media), or NETotic agonists (25 nM PMA; 4 μM A23187; 5 μg/mL lipopolysaccharides (LPS) from *E. coli* 0128; 5 μM Ionomycin) for 120 min. Cells were then fixed, immunostained, and imaged for histone acetylation (H4K5ac) and DNA (DAPI). Cells treated with RPMI show typical polymorphonuclear morphology of neutrophils. When treated with HDACis, belinostat and panobinostat, neutrophils show a further increase in histone acetylation. Blue, DAPI staining for DNA; Magenta, H4K5ac. Scale bar, 14 µm. *n* = 2–3. See Supplementary Figures S1–S3 for single channel confocal images.

**Figure 2:**
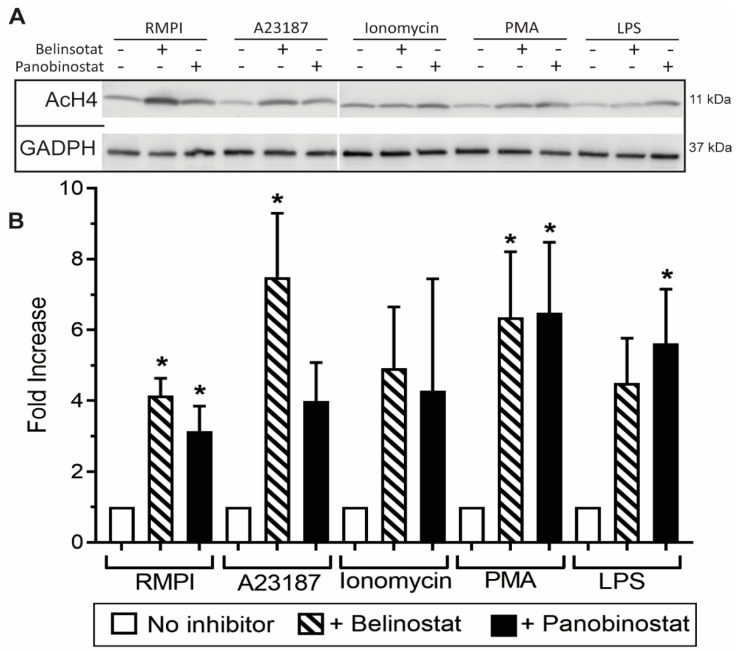
Western blots showing that HDAC inhibitors induce histone acetylation. (**A**) Neutrophils were treated with RPMI (negative control), NETotic agonists (25 nM PMA; 4 μM A23187; 5 μg/mL LPS from *E. coli* 0128; 5 μM Ionomycin) or HDAC inhibitors (250 nM belinostat; 20 nM panobinostat) for 90 min. For each condition, lysates with the same amount of proteins were separated by polyacrylamide gels, proteins were transferred onto a membrane, and specific proteins were immunodetected (GADPH for loading control and H4K5ac for histone acetylation). (**B**) The densitometry analyses show increased histone acetylation when neutrophils are treated with HDAC inhibitors, compared to their corresponding controls. The values were normalized to the respective control values in each experiment. All data are presented as mean ± standard error of the mean (SEM); *n* = 3; *, *p* < 0.05 compared to respective controls. See Supplementary Figure S4 for the full Western blot.

Immunofluorescence images of neutrophils incubated in RMPI (control) were characterized by multi-lobulated nuclei; AcH4 (magenta; Anti-H4K5ac) was barely noticeable (Figure 1). When neutrophils were treated with belinostat or panobinostat, we observed increased levels of AcH4 compared to the untreated controls. It is apparent that acetylated histones are hallmark of neutrophils with NETotic nuclei and decondensing chromatin. Collectively, both immunofluorescence imaging (Figure 1; Supplementary Figures S1–S3; see single colour panels to verify colocalization of AcH4 to chromatin DNA) and immunoblot analyses (Figure 2; see Supplementary Figure S4 for uncropped Western blots) showed that neutrophils cotreated with HDACis and NETotic agonists triggered more AcH4 formation compared to their respective controls without HDACs.

### 3.2. HDAC Inhibitors Promote Baseline NETosis

**Figure 3:**
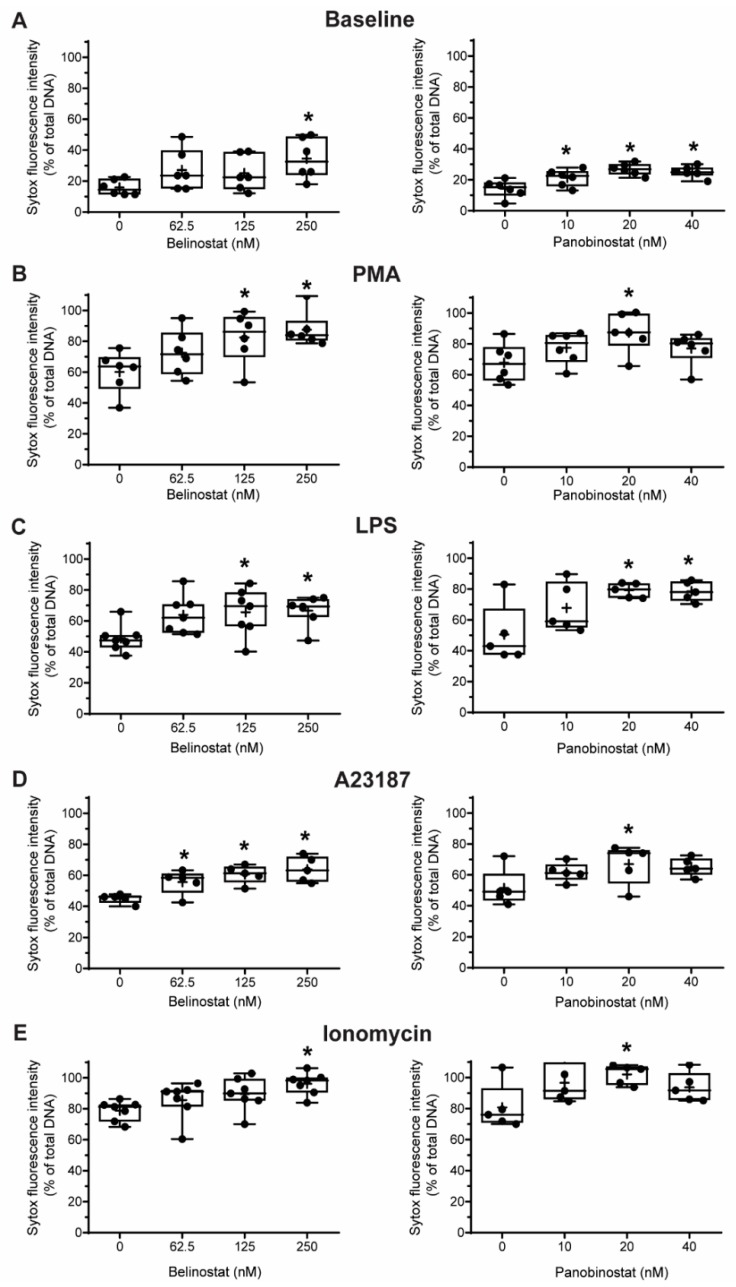
Sytox Green assays suggest that belinostat and panobinostat promote baseline NETosis as well as both NOX-dependent and -independent NETosis. Neutrophils were treated with HDACis and/or NETotic agonists and Sytox Green fluorescence intensities were then measured at 4 h by using a fluorescence plate reader. (**A**) Effects of belinostat and panobinostat on baseline NETosis. (**B**,**C**) Neutrophils were activated with PMA (**B**) or LPS (**C**) in the presence or absence of belinostat or panobinostat. (**D**,**E**) Neutrophils were activated with A23187 (**D**) or ionomycin (**E**) in the presence or absence of belinostat or panobinostat. The full data spread is indicated with lines and boxes are marked with the mean (+), median and upper and lower interquartile ranges. * *p* < 0.05 (One-Way ANOVA with Dunnett post-test, *n* = 5–7). See Supplementary Figure S5 for additional information.

**Figure 4:**
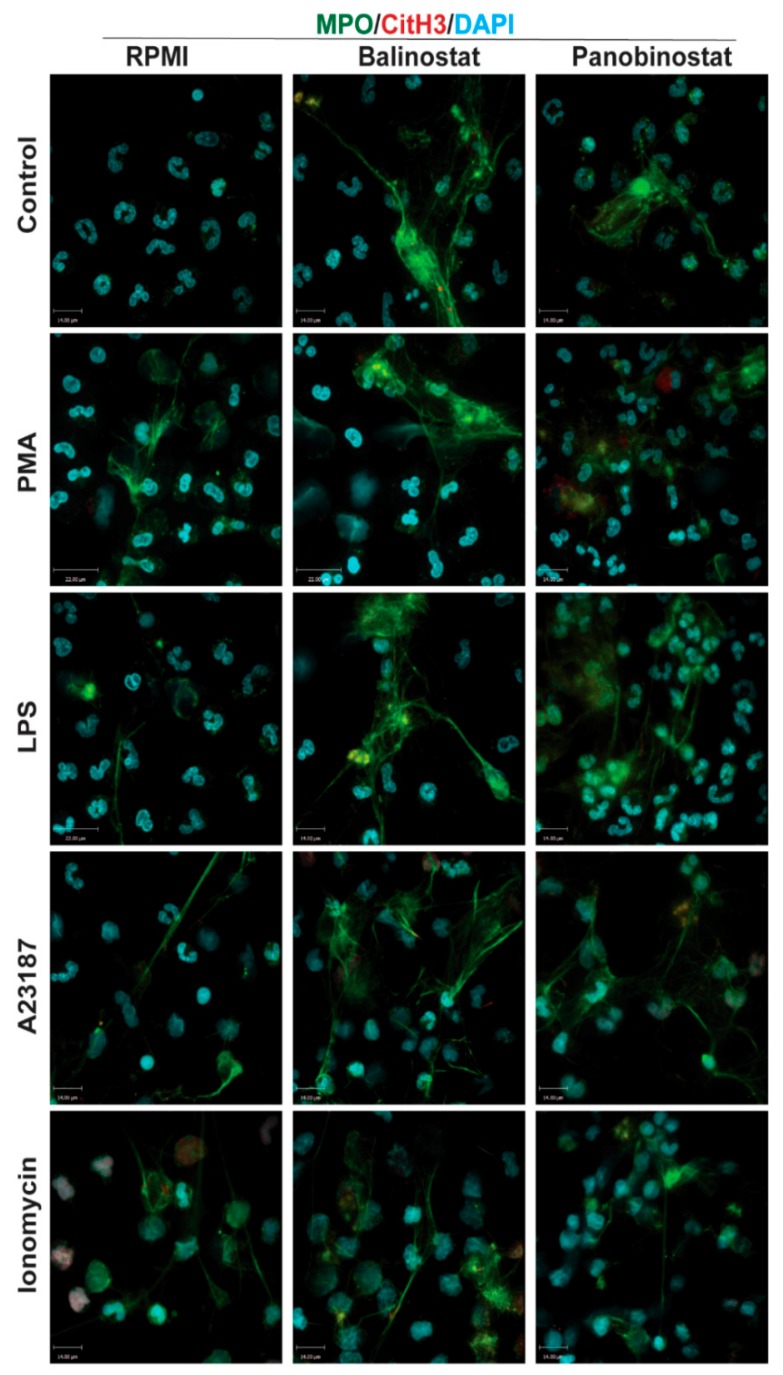
Confocal microscopy images confirm that HDACis promote baseline NETosis, as well as NOX-dependent and -independent NETosis. Neutrophils were treated with negative control (RPMI), or NETotic agonists (25 nM PMA; 4 μM A23187; 5 μg/mL LPS from *E. coli* 0128; 5 μM Ionomycin) for 120 min. Then, cells were fixed, immunostained, and imaged for myeloperoxidase (MPO), citrullinated histone H3 (CitH3) and DNA (DAPI). Cells in RMPI media show typical polymorphonuclear morphology of neutrophils where myeloperoxidase (MPO) can be observed in the cytoplasm. In neutrophils treated with PMA and LPS, MPO co-localizes to NET DNA; limited amounts of CitH3 can be observed. However, CitH3 increases drastically when neutrophils are treated with either A23187 or ionomycin. Interestingly, neutrophils treated with HDACis have increased levels of NETosis in all conditions, which is evident by the intense co-localization of MPO and DNA. Blue: DAPI staining for DNA; Green: MPO; Red: CitH3. Scale bar, 14 µm. *n* = 2–3.

Next, we conducted experiments to determine whether HDACis could mediate NETosis. To determine whether histone acetylation can promote NETosis, we treated neutrophils with pan-HDACis, belinostat and panobinostat, and measured the Sytox Green-stainable DNA as a proxy for % NETosis (% of total DNA); this dye can detect the extracellular DNA, as Sytox Green is cell membrane impermeable. Incubating neutrophils with belinostat showed a gradual increase of Sytox Green accessible DNA, suggesting the increase in NET formation over the 4-h period (Figure 3A). In the presence of 250 nM belinostat, an increase in ~20% of the total DNA above the baseline increase was noted. Similarly, Sytox Green assays showed that the second HDACi, panobinostat also induced cells to undergo NETosis. Over the 4-h treatment period, the presence of either 20 or 40 nM panobinostat significantly increased the levels of NET formation, by ~15% compared to the respective agonist controls (Figure 3A). To verify that the DNA release estimated by Sytox Green is in fact corresponded to NETosis, we performed immunofluorescence assays. MPO colocalizes with DNA during NETosis and considered to be a marker of NETosis [3]. Also, CitH3 was shown to be a marker for calcium-dependent NOX-independent NETosis. Therefore, we used these two makers to verify the NETosis deduced by the Sytox green readings. Images show that extracellular DNA colocalized with MPO and CitH3, confirming that belinostat induces NET formation (Figure 4). The effect of panobinostat on NETosis was also verified by performing immunofluorescence assay, as cells treated with 20 nM panobinostat had increased fluorescence for MPO and CitH3 when compared to the control, and colocalized with DNA (Figure 4).

### 3.3. HDAC Inhibitors Additively Promote NOX-Dependent NETosis

It is well known that PMA and LPS induce NETosis by inducing NOX-derived ROS without the need for CitH3 [8,21,22]. However, it is still unknown whether histone acetylation has any role in mediating NOX-dependent NETosis. Therefore, we pre-incubated neutrophils with either belinostat or panobinostat prior to exposing them to either PMA or LPS for 4 h. Neutrophils treated with PMA had ~45–55% more NETosis than those treated with RPMI alone (Figure 3A,B; Supplementary Figure S5). When pre-treated with belinostat, PMA-induced NETosis was increased in a time- and -concentration-dependent manner, where both 125 and 250 nM belinostat treatments significantly increased DNA release by ~30% at 4-h post-treatment. Similarly, neutrophils cotreated with 20 nM panobinostat for 4 h significantly induced PMA-induced NETosis by ~20% (Figure 3B). NETs released by both belinostat- and panobinostat-activated neutrophils were confirmed by the colocalization of MPO and DNA (Figure 4).

When treated with LPS for 4 h, neutrophils showed a ~35% increase in the NETotic index compared to the baseline control (Figure 3C; Supplementary Figure S5). Plate reader assays demonstrated that both belinostat and panobinostat had an additive effect in increasing LPS-mediated NETosis in a time- and dose-dependent manner (Figure 3C). Total DNA release was ~20% and ~30% higher than LPS-induced NETosis when neutrophils were treated with 250 nM belinostat and 20 nM panobinostat, respectively. These results were confirmed by performing immunofluorescence imaging, where neutrophils treated with either belinostat or panobinostat had increased NETosis with colocalization of MPO and DNA (Figure 4). Therefore, HDACis have additive effects in inducing NOX-dependent NETosis.

### 3.4. HDAC Inhibitors Additively Promote NOX-Independent NETosis

To determine whether histone acetylation promotes NOX-independent NETosis, we pre-incubated neutrophils with either belinostat and panobinostat prior to treating them with either A23187 or ionomycin secreted by Gram-positive bacteria *Streptomyces conglobatus*. Sytox assays showed that calcium ionophore A23187-induced NETosis by ~30–40% above the baseline and at a faster rate than NOX-dependent agonists (Figure 3D; Supplementary Figure S5). Treating neutrophils with belinostat further increased NETosis by ~20% than A23187-induced NETosis (Figure 3D). Similar observations were made when cells were treated with panobinostat, in which it increased NETosis by ~15% more than cells treated with only A23187. Results were confirmed by performing immunofluorescence staining, where increased NETs and colocalization of DNA with MPO and CitH3 were observed when neutrophils were treated with 250 nM belinostat or 20 nM panobinostat (Figure 4).

When neutrophils were treated with another NOX-independent NETosis agonist, ionomycin, the NETosis was increased by ~60–70% than the baseline control (Figure 3E; Supplementary Figure S5). The NETosis further increased by ~20% and ~25% when neutrophils were treated with ionomycin in the presence of belinostat and panobinostat, respectively. NETs released by ionomycin- and HDACis-activated neutrophils were also confirmed by immunofluorescence staining for MPO and DNA (Figure 4). Therefore, HDACis have an additive effect on inducing NOX-independent NETosis.

### 3.5. HDAC Inhibitor Do Not Induce Apoptosis

To determine whether HDACis induce other forms of neutrophil death or cytotoxicity, we examined the nuclear morphology of neutrophils and analyzed an apoptotic marker, cleaved caspase 3 (cCasp-3) [23]. One of the hallmarks of apoptosis is the condensation of nuclei [24]. By analyzing our immunofluorescence images, we found that the neutrophils treated with belinostat or panobinostat show morphological changes that are similar to that of NETotic cells, but not neutrophils undergoing apoptosis (Figure 1 and Figure 4). To confirm this observation, we performed western blots and checked for cleavage of caspase 3 (cCasp-3). A recent study published from our lab showed that neutrophils treated with 0.24 J/cm^2^ ultraviolet irradiation resulted in apoptotic nuclear morphology, but not NETotic morphological changes [25]. Western blotting showed negligible levels of cCasp-3 for neutrophils treated within the IC50 concentrations used in the study for HDACis and/or NETotic agonists, compared to UV-treated neutrophils (Supplementary Figure S6). Therefore, in our experimental conditions with these concentrations, both HDACis promote NETosis, but not apoptosis in neutrophils.

### 3.6. HDAC Inhibitor-Mediated NETosis Requires Baseline ROS

**Figure 5:**
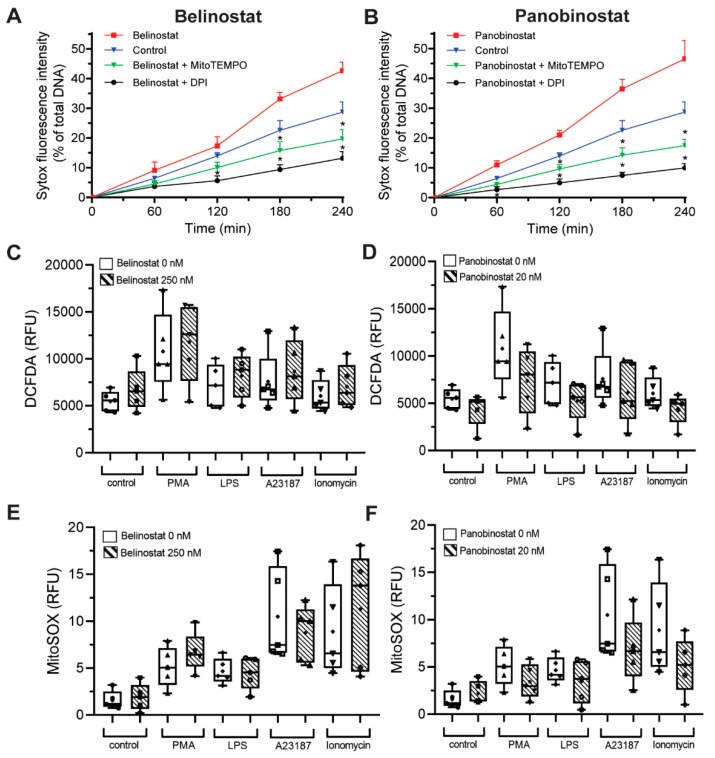
HDACis-mediated NETosis requires ROS production, but these inhibitors do not alter ROS production. Neutrophils were treated with 250 nM belinostat or 20 nM panobinostat in the presence or absence of NOX inhibitor and a mROS scavenger, 1 µM DPI and 200 µM MitoTEMPO, respectively. After 4 h treatment, Sytox Green fluorescence intensities were then measured every 60 min for up to 4 h by using a fluorescence plate reader. (**A**,**B**) Neutrophils cotreated with DPI or MitoTEMPO had significantly lower NETosis than belinostat (**A**) or panobinostat (**B**) treated cells. (**C**–**F**) DHR123 and MitoSOX assays measuring NOX- and mitochondrial-derived ROS production of neutrophils at 30 min, respectively. Neutrophils treated with belinostat (**C**,**E**) or panobinostat (**D**,**E**) do not promote intracellular ROS or mROS levels. Kinetics data are presented as mean ± SEM. In box graphs, full data spread is indicated with lines and boxes are marked with the mean (+), median and upper and lower interquartile ranges. ROS generation data assessed by using DCFDA (**C**,**D**), while by MitoSOX (**E**,**F**). *, *p* < 0.05 (One-Way ANOVA with Tukey post-test conducted at each time points; *n* = 4). See Supplementary Figures S7 and S8 for additional information.

It is known that both NOX-dependent and -independent agonists require ROS to induce NETosis [1]. To determine whether ROS is needed for NETosis induced by histone acetylation, we conducted experiments in the presence of a NOX inhibitor (1 µM DPI) and a mROS scavenger (200 µM MitoTEMPO). When pretreated with DPI or MitoTEMPO, belinostat-induced NETosis was suppressed below baseline (Figure 5A). Both ROS inhibitors were also suppressed panobinostat-induced NETosis (Figure 5B). Therefore, histone acetylation-mediated NETosis requires the presence of baseline ROS.

### 3.7. HDAC Inhibitors Do Not Promote NOX- and Mitochondrial-Derived ROS Production

After establishing that ROS is required to induce HDACi-mediated NETosis, we tested whether HDACis on their own promote ROS production. We incubated neutrophils with HDACis and NETotic agonists and measured the ROS generated by NOX using a DHR123 probe, which becomes fluorescent R123 once oxidized by ROS [1,22]. As expected, DHR123 assays showed that neutrophils treated with PMA and LPS had significantly higher cytosolic ROS levels compared to the control (10 to 90 min post incubation; Figure 5C; Supplementary Figure S7A). As expected, A23187 and ionomycin did not increase ROS levels above baseline control values. Cells treated only with 250 nM belinostat or 20 nM panobinostat also showed no significant increase in cytosolic ROS levels compared to the control (Figure 5C,D; Supplementary Figure S8A). Neutrophils cotreated with HDACis and PMA also had no significant increase in cytosolic ROS production compared to neutrophils treated only with PMA (Figure S8B). Similar observations were made for cells treated with HDACis and LPS, A23187, or ionomycin (Figure S8C–E, respectively). DHR123 results were verified with DCFDA probe as it also measures the cytosolic ROS peroxyl and hydrogen radicals. Cells treated with PMA or LPS, but not A23187 and ionomycin, showed increased ROS levels compared to the control (Figure S7B). Furthermore, HDACis-treated neutrophils had similar RFU values to the controls, even when cotreated with NETotic agonists (Figure S8B–F).

To check whether HDACis induce mROS, MitoSOX assay was performed as this dye fluoresces when it is oxidized by superoxide anion of mitochondrial origin [1]. As expected, neutrophils treated with A23187 and ionomycin showed a significant increase in ROS levels compared to control, starting at 5 min post-treatment (Figure 5E,F; Supplementary Figure S7C). However, cells treated with PMA or LPS did not significantly induce mROS levels. For neutrophils treated only with HDACis, results showed that mROS levels were not significantly higher than the control (Supplementary Figure S8A). Results also demonstrated that HDACis treatments had an insignificant increase of RFU readings when cotreated with either A23187 or ionomycin (Supplementary Figure S8D,E). Similarly, cells cotreated with HDACis and PMA (or LPS) did not show a significant increase in mROS production (Supplementary Figure S8B,C). Therefore, HDACis promote both types of NETosis, without contributing to the production of cytosolic or mROS levels.

### 3.8. Transcriptional Firing Is Required for HDAC Inhibitors to Promote NETosis

Khan and Palaniyar have previously shown that transcriptional firing is required for both NOX-dependent and -independent NETosis; suppression of transcription would inhibit DNA decondensation step even when the early steps of NETosis were occurring, including ROS production [1,3]. Since HDACis induce histone decondensation, we next determined whether HDACi-mediated NETosis require DNA transcription to induce NETosis. To answer this question, we performed Sytox Green assays with neutrophils treated with actinomycin D (Act-D), which binds to destabilized G-C rich promoter regions of DNA and inhibits transcription and incubated them with belinostat or panobinostat for 4 h.

**Figure 6:**
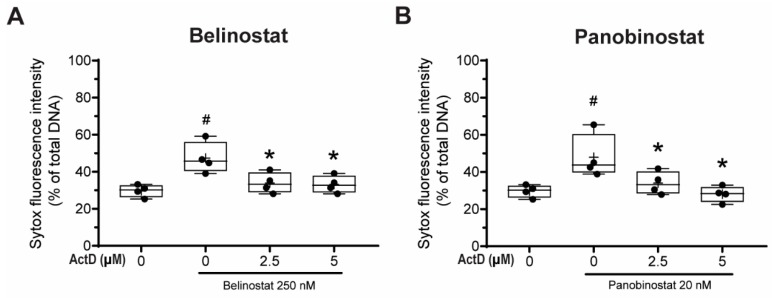
Transcription is required for HDACis to promote NETosis. Neutrophils were treated with 250 nM belinostat or 20 nM panobinostat in the presence of 0, 2.5 and 5 µM actinomycin D (Act-D), a DNA transcription inhibitor. After 4-h treatment, Sytox Green fluorescence intensities were measured by using a fluorescence plate reader. (**A**,**B**) Neutrophils treated with Act-D had significantly lower total DNA release than belinostat- (**A**) or panobinostat-induced NETosis (**B**). The full data spread is indicated with lines, and boxes marked with the mean (+), median and upper and lower interquartile ranges. * *p* < 0.05 versus baseline with no HDACi; # *p* < 0.05 versus HDACi only (One-Way ANOVA with Tukey post-test conducted at each time points; *n* = 4). See Supplementary Figure S9 for additional information.

Neutrophils treated with 250 nM belinostat had a significant increase in NETosis compared with the control (Figure 6A). By contrast, the NETosis was significantly inhibited when cells were pre-treated with 2.5 and 5 µM Act-D in both time-dependent and dose-dependent manner (Supplementary Figure S9A). Similar results were found when neutrophils were co-treated with both 20 nM panobinostat and Act-D (Figure 6B; Supplementary Figure S9B). Therefore, HDACi-mediated increase in NETosis involves transcription.

## 4. Discussion

**Figure 7:**
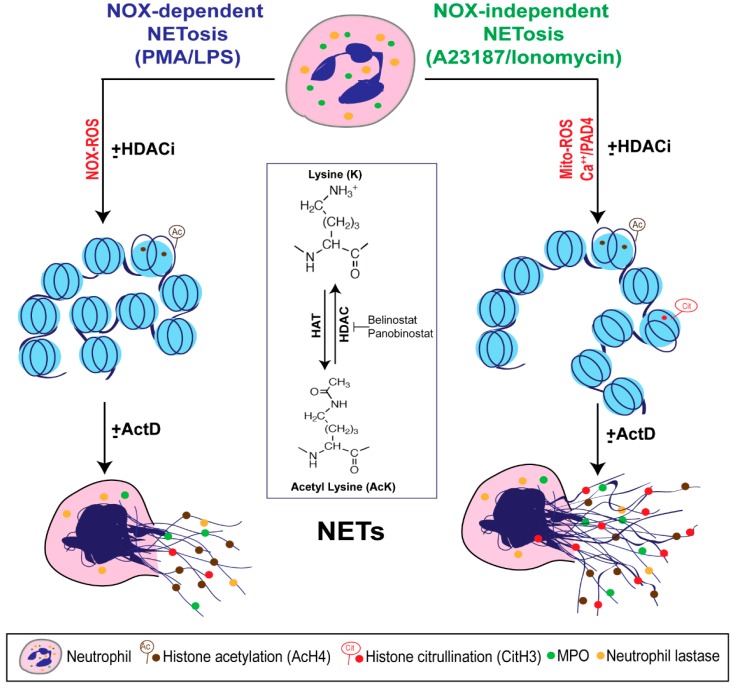
A unifying model showing the role of histone acetylation in NETosis. PMA and LPS induce nicotinamide adenine dinucleotide phosphate (NADPH) oxidase (NOX)-dependent whereas calcium ionophores such as A23187 and ionomycin activate NOX-independent pathways of NETosis. NOX-dependent and -independent pathways exert their downstream effects by inducing NOX ROS and mitochondrial ROS, respectively. After several intermediate steps, chromatin decondenses to become NETs. In the resting neutrophil nuclei, negatively charged DNA wraps around highly positively charged histones (e.g., amino acids arginine and lysine) to form tightly compacted chromatin. Addition of an acetyl group (H_3_C-C=O) to the tip of the side chain (-NH_3_) of the N-terminal lysines of histones (e.g., H4 histone with K5 acetylation; H4K5ac, or AcH4) eliminates the positive charge (middle inset). Hence, histone acetylation weakens the overall chromatin structure, and particularly uncompacts the nucleosomes at promoter regions to allow the entry of various proteins to access DNA (e.g., transcription machinery). Histone acetyl transferases (HAT) add acetyl groups to lysine residues whereas histone deacetylases (HDACs; 18 in neutrophils) remove the acetyl groups. A balance between HAT and HDAC activities determines the degree of histone acetylation. HDAC inhibitors (HDACis; e.g., Belinostat, Panobinostat) shift the balance towards increased acetylation of histones. This modification promotes baseline as well as NOX-dependent and -independent NETosis. Since HDACis modify histones that are downstream of ROS production, they do not affect ROS production during NETosis. During the induction of calcium ionophore-mediated NOX-independent pathway, increase in intracellular calcium concentrations also enables the cytoplasmic peptidylarginine deiminase 4 (PAD4) to translocate into the nuclei. This active form of PAD4 (PAD4:Ca^2+^ complex) citrullinates the histones (i.e., the removal of the positive charge from arginine; e.g., CitH3) particularly at the promoter regions. This modification also facilitates transcription. Process of genome-wide transcription is necessary for decondensation of chromatin for both types of NETosis. In summary, acetylation of histones promotes transcription and subsequent chromatin decondensation during baseline NETosis as well as both NOX-dependent and NOX-independent NETosis.

Several studies focused on citrullination of histone and considered that CitH3 formation is an important contributor to NETosis. Our previous studies showed that histone citrullination is a hallmark of calcium-mediated NOX-independent, but not NOX-dependent NETosis [3,8]. However, the role of histone acetylation on NETosis was unknown [11,12]. In this study, we demonstrated that histone hyperacetylation is found in NETotic cells that are stimulated by both NOX-dependent and -independent agonists, and more pronounced when neutrophils are treated with HDACis, either belinostat or panobinostat (Figure 1 and Figure 2). Both belinostat and panobinostat induce NETosis and additively promote NOX-dependent and NOX-independent NETosis (Figure 3 and Figure 4). Our results show that HDACis require baseline ROS production (Figure 5), but they do not induce NOX- or mitochondria-derived ROS production (Figure 5; Supplementary Figure S8). In addition, we demonstrated that HDACis-mediated NETs formation also uses transcriptional firing to decondense chromatin (Figure 6). Collectively, these data show that histone acetylation promotes baseline NETosis as well as both NOX-dependent and -independent NETosis (Figure 7).

Our study provides evidence that histone acetylation promotes NETosis. By performing immunofluorescent imaging, we found that belinostat and panobinostat both induce histone acetylation as evident by H4K5ac fluorescent intensities and Western bots (Figure 1 and Figure 2, respectively). Interestingly, we also found that histone acetylation increases as part of NOX-dependent and -independent NETosis. This finding suggests that histone acetylation-deacetylation balance is altered during both types of NETosis. Therefore, unlike histone citrullination [3], histone acetylation could promote both types of NETosis (Figure 3). To further understand the mechanism, we first used two ROS probes, DHR123 and DCFDA, to measure most of the cytosolic ROS [26]. Consistent with our previous studies [1,8,9], we found baseline increase in ROS in neutrophils and a substantial increase of cytosolic ROS levels during the activation of NOX pathway (e.g., with PMA, LPS), mROS increase during the activation of NOX-independent pathway (e.g., with A23187 and ionomycin; Figure 5; Supplementary Figure S7). However, histone acetylation does not significantly alter neither NOX nor mitochondrial ROS production (Supplementary Figure S8). This is consistent with the fact that histone acetylation is a downstream modification, and hence, should not affect ROS production.

Khan and Palaniyar (2017) have shown that transcriptional firing is required for NETosis to occur [3]. Since histone acetylation could induce local chromatin decondensation and facilitate transcription initiation for further decondensation, we tested the effect of histone acetylation on transcription. Act-D inhibits acetylation induced NETosis (Figure 6); hence, we propose that acetylation is another modification that promotes baseline as well as agonist-induced NET formation via transcription. Hollands et al. (2016) attempted to study the effect of a histone acetyltransferase (HAT) inhibitor, anacardic acid, on NETosis [27]. However, anacardic acid has off-target effects and significantly induces intracellular ROS production, similar to PMA [28,29,30,31,32]. Therefore, the effect of histone acetylation cannot be directly discerned by anacardic acid; inhibitors without off-target effects are required to elucidate the role of histone acetylation on NETosis using this approach.

An increase in ~20–30% baseline, or the agonist-induced release of modified autoantigen from neutrophils could contribute significantly to autoimmune diseases. Two studies examined the role of post-translational modification of NET histones in Lupus. Liu et al. (2012) demonstrated that treating neutrophils, isolated from systemic lupus erythematosus (SLE) peripheral blood, with PMA, ionomycin and LPS can induce autoantibodies that targeted NETs [33]. Careful analysis revealed that certain histone modifications, especially H2Bac, induced autoreactivity. Another recent study showed that NETs generated from SLE patients had more acetylated histones than the NETs generated from healthy donors [34]. Therefore, NETs generated with different forms of acetylation could affect autoimmune diseases (e.g., SLE) or clinical conditions in which these HDAC inhibitors are used (e.g., cancer treatments) [19,20].

In summary, histone acetylation occurs during both types of NETosis, and HDACis increase AcH4 formation in primary neutrophils. HDACis increase baseline NETosis and have an additive effect on both NOX-dependent and -independent NETosis. Baseline histone acetylation-mediated NETosis requires ROS production, but acetylation does not alter ROS production in neutrophils at baseline or during agonist-induced NETosis. This post-translational modification of histone promotes NETosis via facilitating transcription-mediated chromatin decondensation step. We conclude that histone hyperacetylation promotes NETosis (Figure 7).

## Supplementary Materials

The following are available online at http://www.mdpi.com/2218-273X/9/1/32/s1, Figure S1: Single channel confocal microscopy images showing histone acetylation in the absence of HDAC inhibitors; Figure S2: Single channel confocal microscopy images showing that belinostat promotes histone acetylation; Figure S3: Single channel confocal microscopy images showing that panobinostat promotes histone acetylation; Figure S4: Western blots confirming HDAC inhibitors’ potential to induce histone acetylation; Figure S5: Sytox Green assays comparing the levels of NETosis of PMA, LPS, A23187 and ionomycin with the baseline; Figure S6: Western blots showing HDAC inhibitors do not lead to apoptosis; Figure S7: DHR123, DCFDA and MitoSOX assays for neutrophils treated with NOX-dependent and -independent agonists; Figure S8: HDACis do not promote NOX- and mitochondrial-derived ROS production; Figure S9: Transcription is required for HDACis-mediated NETosis.
