# Supplementary material for: Histone Acetylation Promotes Neutrophil Extracellular Trap Formation
Source: Biomolecules. 2019 Jan 18;9(1):32. doi: 10.3390/biom9010032 (PMC6359456; doi:10.3390/biom9010032)
Supplement: Supplementary file 1 [file biomolecules-09-00032-s001.pdf]

biomolecules

Supplementary Information

Histone Acetylation Promotes Neutrophil Extracellular Trap Formation

Hussein J. Hamam [1,2], Meraj A. Khan [1,2] and Nades Palaniyar [1,2,3,*]

[1] Program in Translational Medicine, Peter Gilgan Centre for Research and Learning, The Hospital for Sick Children, Toronto, ON M5G 0A4, Canada; hussein.hamam@sickkids.ca (H.J.H); meraj.khan@sickkids.ca (M.A.K)
[2] Department of Laboratory Medicine and Pathobiology, Faculty of Medicine, University of Toronto, Toronto, ON M5S 1A8, Canada
[3] Institute of Medical Sciences, Faculty of Medicine, University of Toronto, Toronto, ON M5S 1A8, Canada
* Correspondence: nades.palaniyar@sickkids.ca

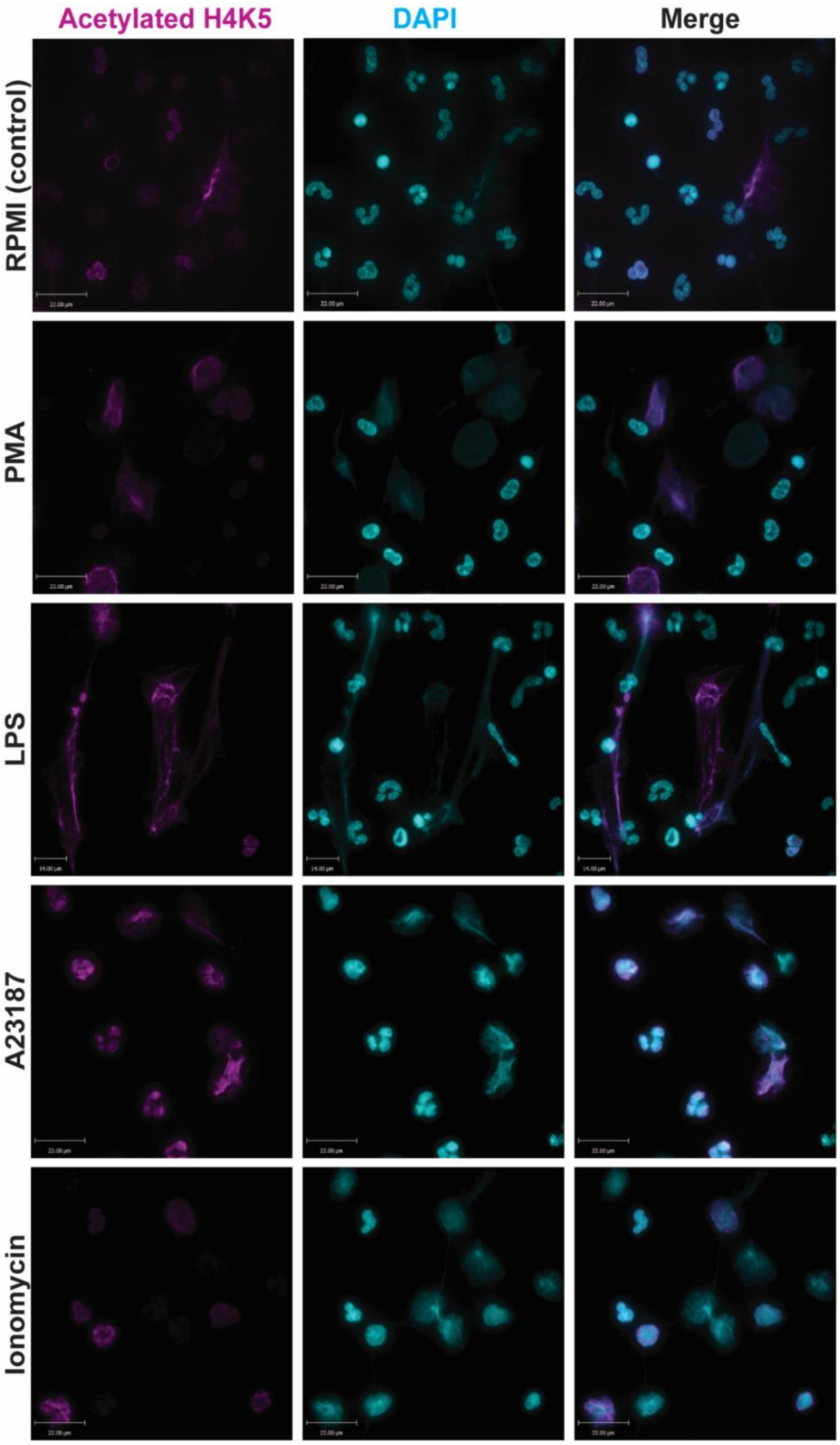

Figure S1. Single channel confocal microscopy images showing histone acetylation in the absence of HDAC inhibitors. Neutrophils were treated with the negative control (RPMI), or NETotic agonists (25 nM PMA; 4 µM A23187; 5 µg/ml LPS from E. coli 0128; 5 µM Ionomycin) for 120 min. Cells were then fixed, immunostained, and imaged for histone acetylation (H4K5ac) and DNA (DAPI). Cells treated with RMPI show typical polymorphonuclear morphology of neutrophils. Blue, DAPI staining for DNA; Magenta, H4K5ac. Scale bar, 14 µm. *n* = 2-3.

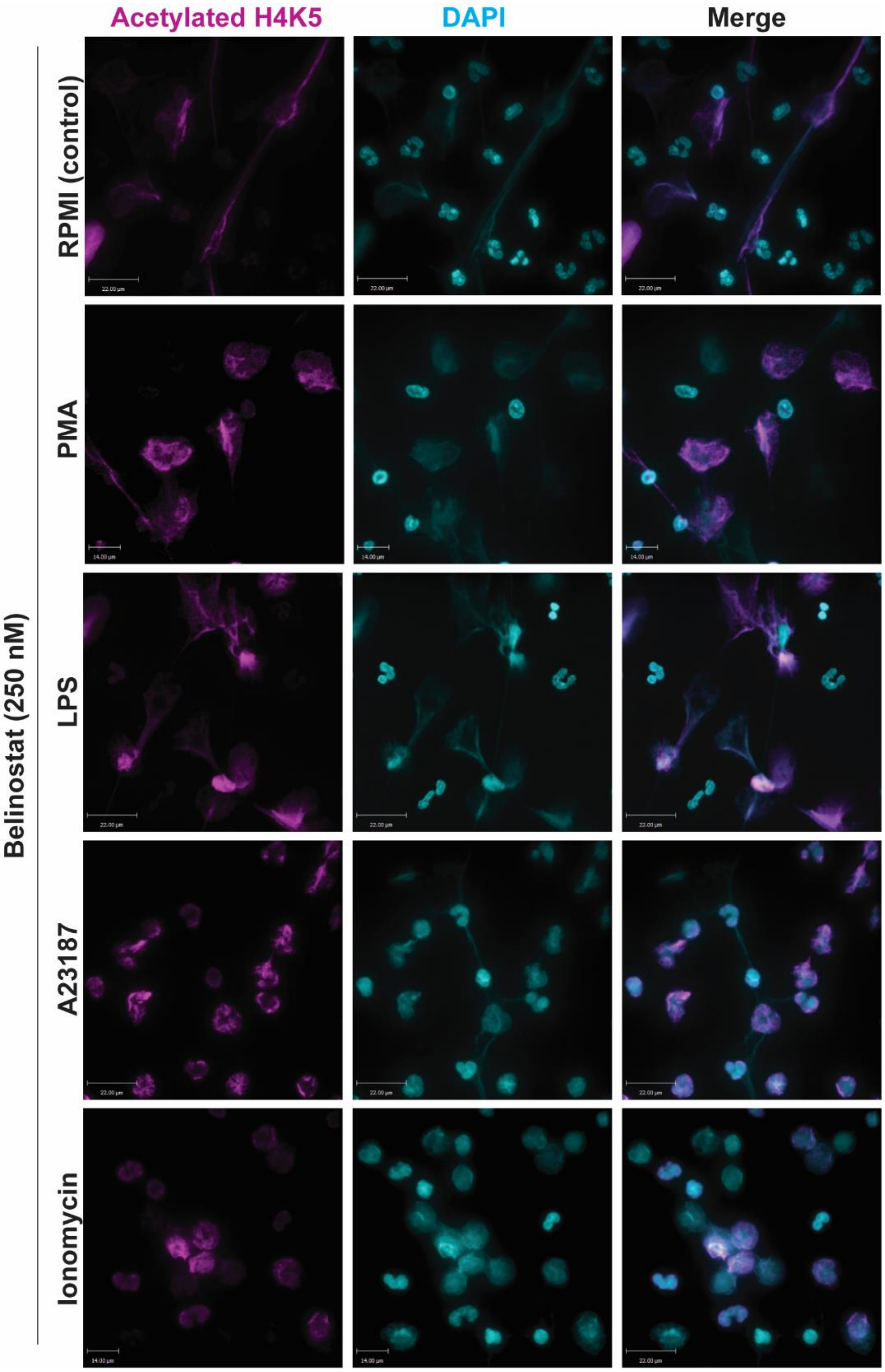

Figure S2. Single channel confocal microscopy images showing that belinostat promotes histone acetylation. Neutrophils were treated with the negative control (RPMI), NETotic agonists (25 nM PMA; 4 μM A23187; 5 μg/ml LPS from *E. coli* 0128; 5 μM Ionomycin) or HDAC inhibitor (250 nM belinostat) for 150 min. Cells were then fixed, immunostained, and imaged for histone acetylation (H4K5ac) and DNA (DAPI). Cells treated with RMPI show typical polymorphonuclear morphology of neutrophils. In neutrophils treated with belinostat, there is a noticeable increase in immunostaining of H4K5ac. Blue, DAPI staining for DNA; Magenta, H4K5ac. Scale bar, 14 μm. *n* = 2-3.

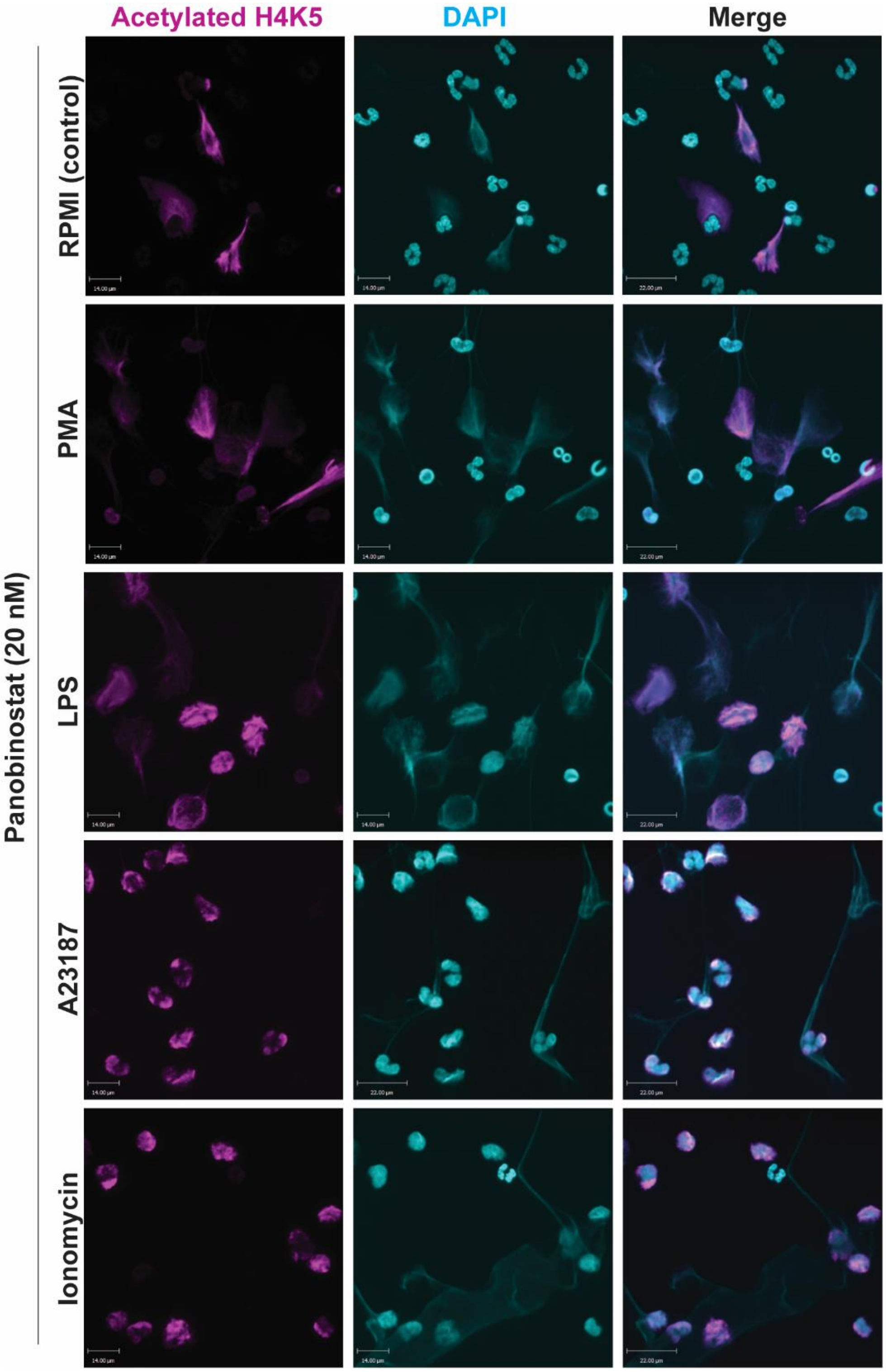

Figure S3. Single channel confocal microscopy images showing that panobinostat promotes histone acetylation. Neutrophils were treated with the negative control (RPMI), NETotic agonists (25 nM PMA; 4 μM A23187; 5 μg/ml LPS from *E. coli* 0128; 5 μM Ionomycin) or HDAC inhibitors (20 nM panobinostat) for 150 min. Cells were then fixed, immunostained, and imaged for histone acetylation (H4K5ac) and DNA (DAPI). Cells treated with RMPI show typical polymorphonuclear morphology of neutrophils. In neutrophils treated with panobinostat, there is a noticeably increase in immunostaining of H4K5ac. Blue, DAPI staining for DNA; Magenta, H4K5ac. Scale bar, 14 μm. n = 2-3.

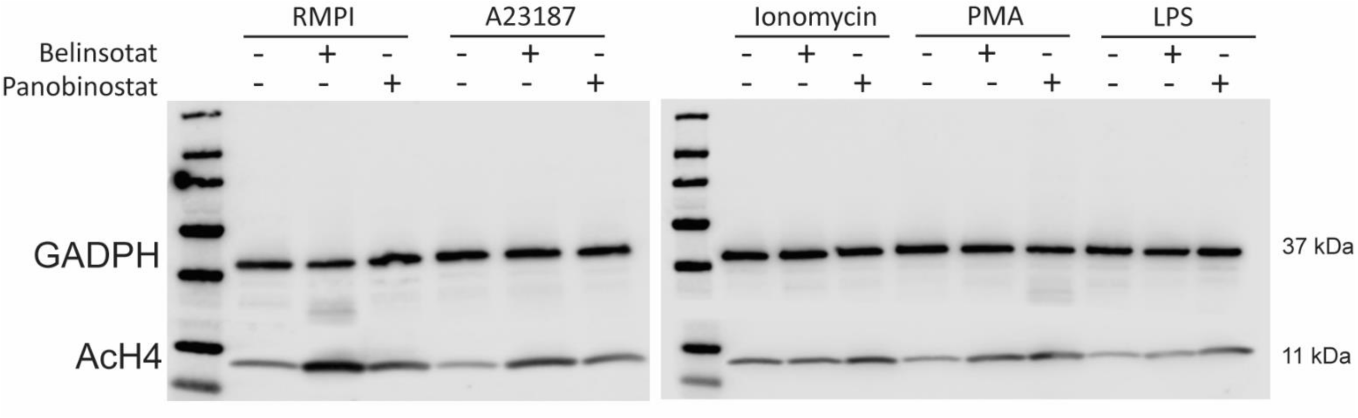

Figure S4. Western blots confirming HDAC inhibitors' potential to induce histone acetylation. Neutrophils were treated with RPMI (negative control), NETotic agonists (25 nM PMA; 4 μM A23187; 5 μg/ml LPS from *E. coli* 0128; 5 μM Ionomycin) or HDAC inhibitors (250 nM belinostat; 20 nM panobinostat) for 90 min. RIPA buffer and DNAse were used and then sonicated to prepare whole cell lysates. For each condition, 25 μg/ml lysates were separated by using 4-20% precast protein gels. Overnight staining for GADPH (loading control) and histone acetylation (H4K5ac) shows increased histone acetylation when neutrophils are treated with HDAC inhibitors, compared to their corresponding controls. Images are representative of 3-4 independent experiments. See Fig. 2 for densitometry analysis.

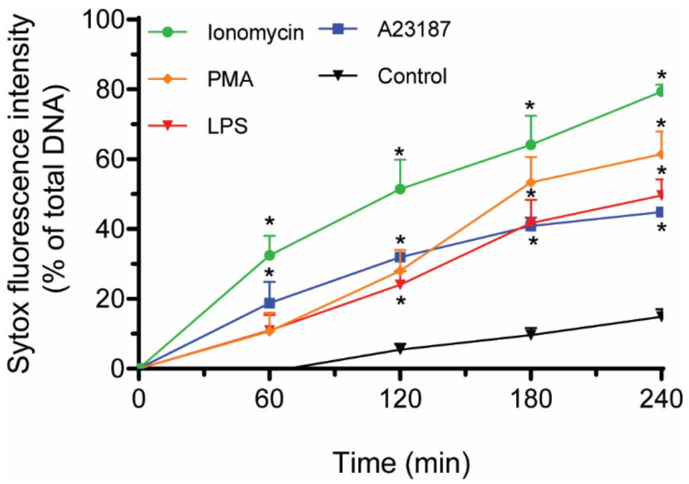

Figure S5. Sytox Green assays comparing the levels of NETosis of PMA, LPS, A23187 and ionomycin with the baseline. Neutrophils were treated with NETotic agonists (25 nM PMA; 4 μM A23187; 5 μg/ml LPS from *E. coli* 0128; 5 μM Ionomycin) and Sytox Green fluorescence intensities were then measured every 60 min for up to 4 h by using a fluorescence plate reader. Cells treated with ionomycin or A23187 show a significant increase in NETotic index starting at 60 min post-treatment, while PMA- and LPS-stimulated neutrophils significantly induce NETosis starting from 120 min post-treatment. All data are presented as mean ± SEM; *, $p < 0.05$ (One-Way ANOVA with Dunnett post-test conducted at each time points, comparing with untreated controls; $n = 5$).

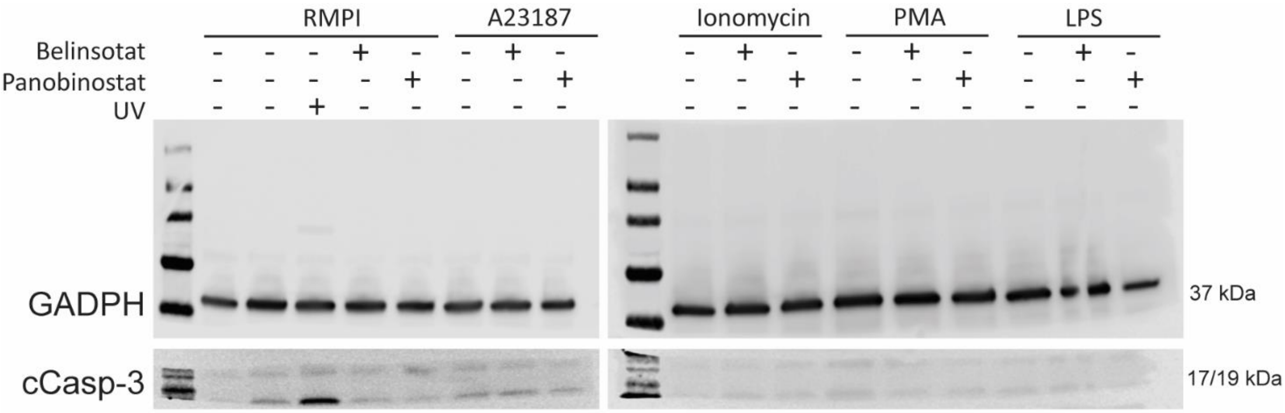

Figure S6. Western blots showing HDAC inhibitors do not lead to apoptosis. Neutrophils were treated with RPMI (negative control), UV (positive control; 0.24 J/cm^2), NETotic agonists (25 nM PMA; 4 μM A23187; 5 μg/ml LPS from *E. coli* 0128; 5 μM Ionomycin) or HDAC inhibitors (250 nM belinostat; 20 nM panobinostat) for 90 min. RIPA buffer and DNAse were used and then sonicated to prepare whole cell lysates. For each condition, 25 μg/ml lysates were separated by using 4-20% precast protein gels. Overnight staining for GADPH (loading control) and cleaved caspase 3 (cCasp-3) shows HDAC inhibitors do not induce neutrophils to undergo apoptosis. See Figs. 1, 4 for cell morphology.

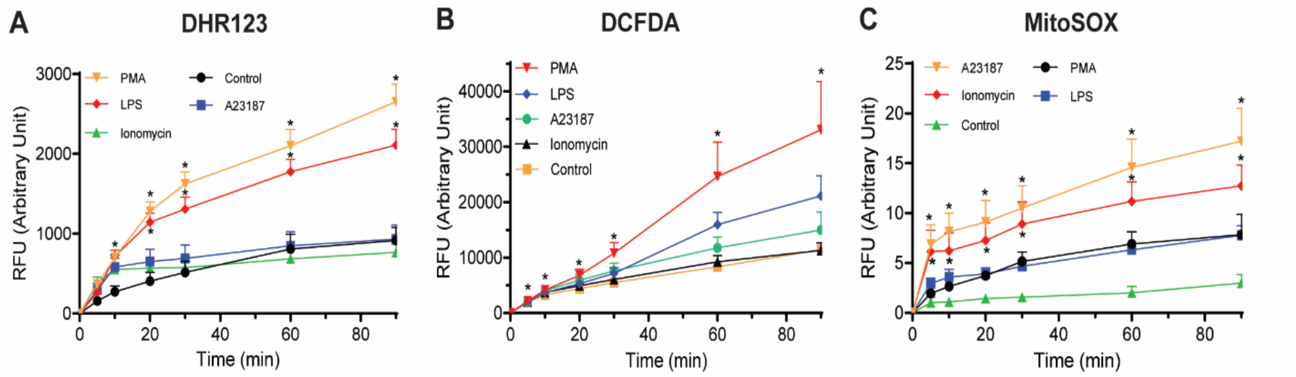

Figure S7. DHR123, DCFDA and MitoSOX assays for neutrophils treated with NOX-dependent and -independent agonists. (A, B) DHR123 (A) and DCFDA (B) assays measuring NOX-mediated ROS production of neutrophils. (C) MitoSOX assay measuring mROS levels. Cells treated with PMA or LPS have increased levels of cytosolic ROS, but not mROS. Induced levels of mROS are found for neutrophils treated with A23187 or ionomycin. All data are presented as mean ± SEM; *, $p < 0.05$ (One-Way ANOVA with Dunnett post-test conducted at each time points; n = 4-5).

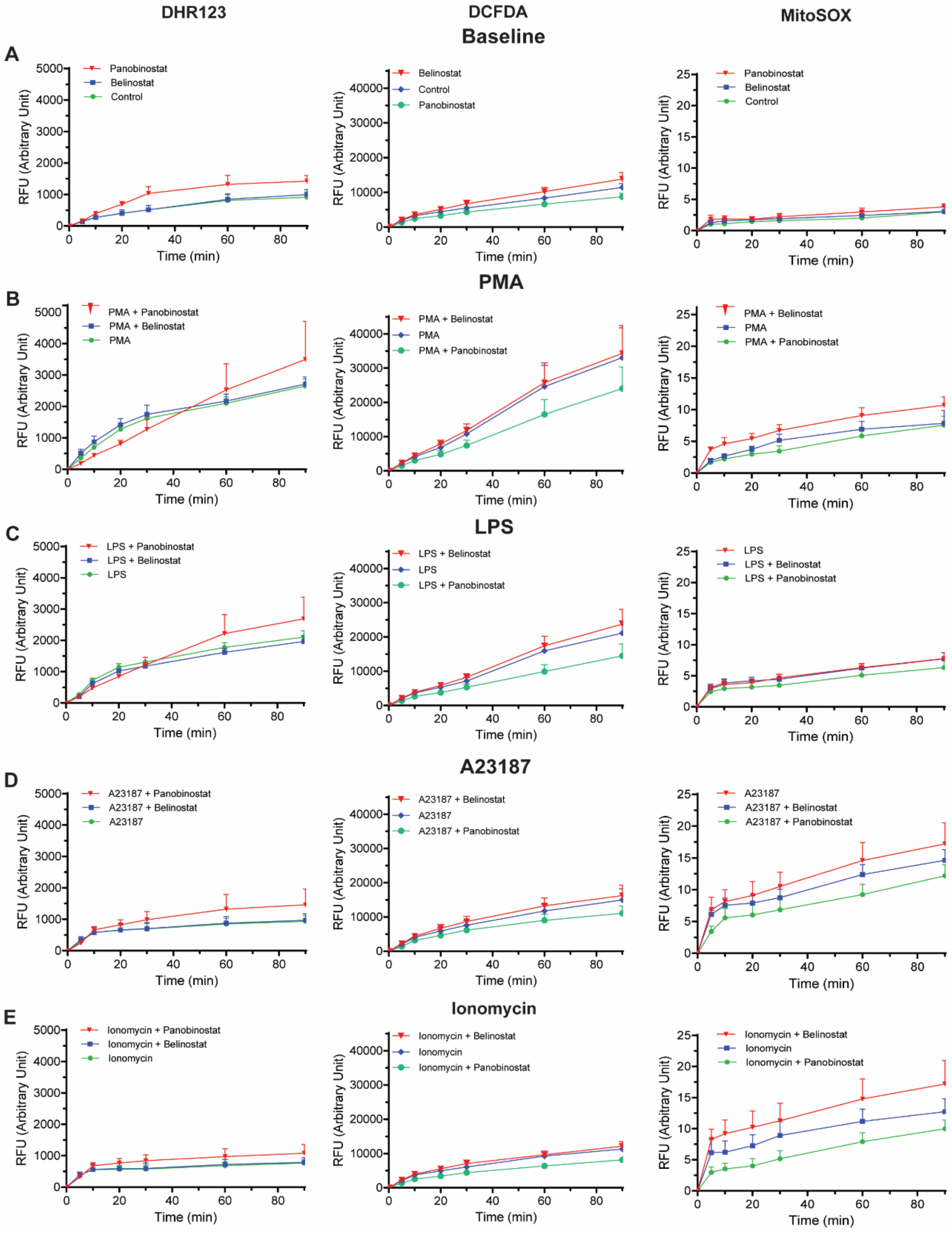

Figure S8. HDACis do not promote NOX- and mitochondrial-derived ROS production. DHR123 and DCFDA assays measuring NOX-mediated ROS production of neutrophils. MitoSOX assay measuring mROS levels. (A) Neutrophils treated with belinostat (250 nM) or panobinostat (20 nM) do not have increased levels of intracellular ROS. (B, C) Cells treated with PMA (B) or LPS (C) have increased levels of cytosolic ROS, but not mROS. (D, E) Induced levels of mROS are found for neutrophils treated with A23187 (D) or ionomycin (E). Neutrophils cotreated with HDACis and NETotic agonists do not show increased levels of ROS. All data are presented as mean ± SEM; *, $p < 0.05$ (One-Way ANOVA with Dunnett post-test conducted at each time points; n = 4-5).

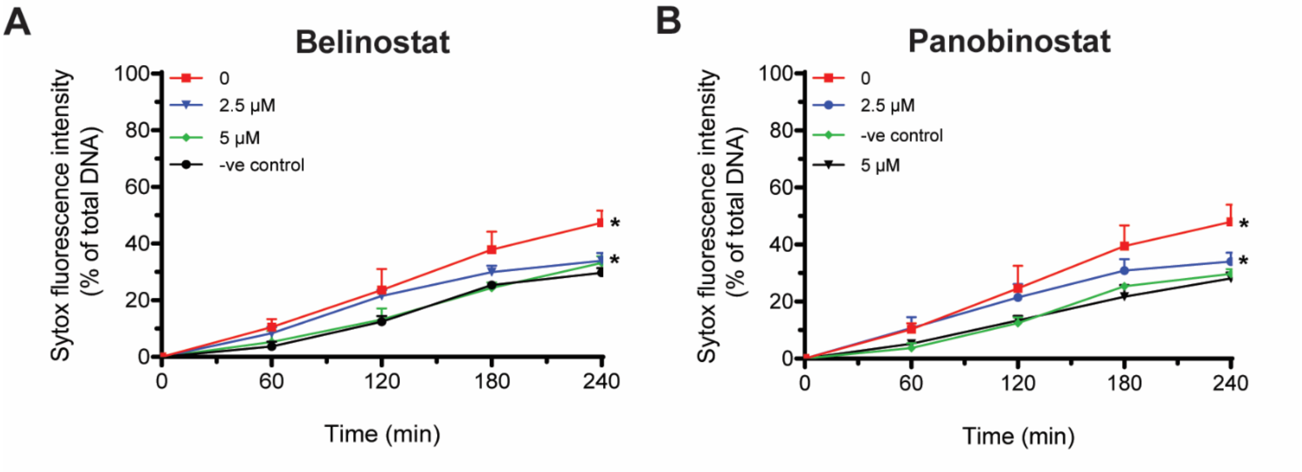

Figure S9. Transcription is required for HDACis-mediated NETosis. Neutrophils were treated with 250 nM belinostat or 20 nM panobinostat in the presence of 0, 2.5 and 5 µM actinomycin D (Act-D), a DNA transcription inhibitor. After 4 h treatment, Sytox Green fluorescence intensities were measured every 60 min for 4 h by using a fluorescence plate reader. (A, B) Neutrophils treated with Act-D had significantly lower total DNA release than belinostat- (A) or panobinostat-induced NETosis (B). All data are presented as mean ± SEM; *, $p < 0.05$ (One-Way ANOVA with Tukey post-test conducted at each time points; $n = 4$).
